# In Vitro Effects of Mitochondria-Targeted Antioxidants in a Small-Cell Carcinoma of the Ovary of Hypercalcemic Type and in Type 1 and Type 2 Endometrial Cancer

**DOI:** 10.3390/biomedicines10040800

**Published:** 2022-03-29

**Authors:** Mariana Castelôa, Beatriz Moreira-Pinto, Sofia Benfeito, Fernanda Borges, Bruno M. Fonseca, Irene Rebelo

**Affiliations:** 1 CIQUP/Department of Chemistry and Biochemistry, Faculty of Sciences, University of Porto, Rua do Campo Alegre 1021/1055, 4169-007 Porto, Portugal; mariana15cas@gmail.com (M.C.); ester.benfeito@fc.up.pt (S.B.); fborges@fc.up.pt (F.B.); 2 UCIBIO, Applied Molecular Biosciences Unit, Laboratory of Biochemistry, Faculty of Pharmacy, University of Porto, Rua de Jorge Viterbo Ferreira 228, 4050-313 Porto, Portugal; abeatrizmoreira.pinto@gmail.com; 3 Associate Laboratory i4HB, Laboratory of Biochemistry, Institute for Health and Bioeconomy, Faculty of Pharmacy, University of Porto, Rua de Jorge Viterbo Ferreira 228, 4050-313 Porto, Portugal

**Keywords:** cancer, ovary, endometrium, anticancer agents, mitocans, TPP, antioxidants

## Abstract

Small-cell carcinoma of the ovary of hypercalcemic type (SCCOHT) and endometrial cancer from type 1 and type 2 are gynecological tumors that affect women worldwide. The treatment encompasses the use of cytotoxic drugs that are nonspecific and inefficient. “Mitocans”, a family of drugs that specifically target tumor cells’ mitochondria, might be a solution, as they conjugate compounds, such as antioxidants, with carriers, such as lipophilic cations, that direct them to the mitochondria. In this study, caffeic acid was conjugated with triphenylphosphonium (TPP), 4-picolinium, or isoquinolinium, forming 3 new compounds (Mito6_TPP, Mito6_picol., and Mito6_isoq.) that were tested on ovarian (COV434) and endometrial (Hec50co and Ishikawa) cancer cells. The results of MTT and neutral red assays suggested a time- and concentration-dependent decrease in cell viability in all tumor cell lines. The presence of apoptosis was indicated by the Giemsa and Höechst staining and by the decrease in mitochondrial membrane potential. The measurement of intracellular reactive oxygen species demonstrated the antioxidant properties of these compounds, which might be related to cell death. Generally, Mito6_TPP was more active at lower concentrations than Mito6_picol. or Mito6_isoq., but was accompanied by more cytotoxic effects, as shown by the lactate dehydrogenase release. Non-tumorous cells (HFF-1) showed no changes after treatment. This study assessed the potential of these compounds as anticancer agents, although further investigation is needed.

## 1. Introduction

Ovarian cancer is the third most common cancer of the female reproductive tract and the most lethal in this category [1,2]. In particular, small-cell carcinoma of the ovary of hypercalcemic type (SCCOHT) is a rare and extremely aggressive tumor that affects mostly women younger than 40 years [3]. Although endometrial cancer is not as deadly as ovarian cancer, it is estimated to be the second most common gynecological cancer [1,2,4]. It can be classified into well-differentiated types: type 1, which generally presents a good prognosis and requires estrogen to grow, or type 2, which is estrogen-independent and consists of poorly differentiated cells, associated with endometrial atrophy and a worse prognosis [5,6]. There is a need to develop new anticancer agents that can damage these tumors effectively, as SCCOHT is extremely lethal and endometrial tumors have a high incidence that is expected to increase [2]. The current treatment for these pathologies is based mostly on cytotoxic drugs, such as cisplatin, carboplatin, and paclitaxel, that also produce aggressive side effects [7,8]. Accordingly, the aim at the moment is to develop targeted drugs that can substitute the cytotoxic drugs by attacking specific molecular targets, barely affecting healthy cells, and therefore causing fewer side effects and avoiding drug resistance [9]. An example is the effort being made to optimize the delivery of doxorubicin to the mitochondria; for instance, either through the use of micelles [10] or through the use of a lipophilic cation carrier [11].

The doxorubicin conjugated with the lipophilic cation [11] is part of a family of compounds that are being studied with the aim of improving the available cancer treatments—the mitocans—which are characterized by their ability to specifically target tumor cells’ mitochondria [12]. These compounds are showing potential, since they use the different features of tumor cells’ mitochondria compared to healthy cells to differentiate them. Some aspects of tumor cells that allow their specific attack by the mitocans include an aberrant metabolism, characterized by an unbalance between oxidative phosphorylation and glycolysis, a different mitochondrial membrane potential, and a higher production of reactive oxygen species (ROS) [13,14,15]. At the same time, tumor cells’ mitochondria still maintain important functions in tumor progression, such as redox control and transcription regulation [13,16,17]. Because of that, the mitochondria are promising targets to use as a base for effective treatment, regardless of the patient, as the same type of tumor has different characteristics in different patients, implying that a drug that affects only one pathway may not be effective in all cases [18,19]. However, the mitochondrial target of tumor cells might be more effective in those tumors that are highly dependent on the mitochondrial metabolism, such as small-cell lung cancer, ovarian cancer, breast cancer, and acute myeloid leukemia [20].

**Figure 1:**
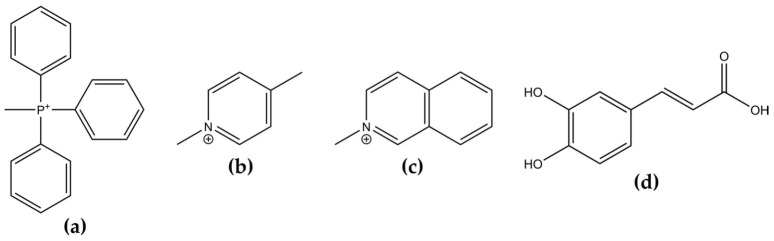
Chemical structures of (**a**) Triphenylphosphonium; (**b**) 4-Methylpyridinium or 4-picolinium; (**c**) Isoquinolinium; and (**d**) Caffeic acid.

The mitocans are generally composed of a small bioactive molecule, such as an antioxidant, and a mitochondria-oriented motif, the most common being a lipophilic cation [14,21]. However, the use of antioxidants to combat cancer is still a debatable topic [22]. It is expected that the antioxidants capture the excess ROS that are essential to tumor survival, and therefore, antioxidants may block tumor progression [23,24]. There are no consistent studies regarding the role of antioxidants against tumors, but some researchers predict that the failures detected may be due to their lack of specificity [25]. Recent studies even suggest that using antioxidants associated with mitochondria-targeted lipophilic cation carriers might be a strategy to increase their pharmacological effect against cancer [26,27] and other pathologies [28]. One of the most-used carriers is the triphenylphosphonium (TPP) cation (Figure 1a) that has been used to target the mitochondria, as it can accumulate in its matrix due to its negative membrane potential [27,29]. Moreover, it has a higher tendency to accumulate in tumor cells as their mitochondrial membrane potential is around 60 mV more negative than other cells; however, this does not exclude the fact that it can also accumulate in non-tumorous cells, although in lower doses [14]. It has shown intrinsic cytotoxic activity, but it is usually conjugated with other compounds to improve their effectiveness in a variety of pathologies [14,30,31]. It was demonstrated, for example, that several TPP-conjugated decyl-polyhydroxybenzoates were cytotoxic and inhibited the metastatic capacity of breast cancer cell lines [32]. Another example is the pyridine derivatives, such as 4-picolinium (Figure 1b), that demonstrated that its conjugation with, for example, Pt(II)(hydrazone) [33] or cisplatin [34] had higher cytotoxicity than the original compounds in cancer cell lines. The isoquinoline derivatives (Figure 1c) have also been conjugated with other compounds, and have shown the ability to inhibit cancer-associated enzymes [35]. Moreover, it was observed that isoquinoline-based compounds were more cytotoxic to tumor cells than to other cells [36].

In particular, derivatives of the natural antioxidant caffeic acid (Figure 1d) linked to the TPP cation were developed with the aim of preventing the neurodegeneration and aging processes [37,38]. One of these compounds, here called Mito6_TPP, showed high toxicity and cell proliferation inhibition in various cell lines isolated from tumors [38,39]. Even though the authors were looking for compounds able to prevent mitochondrial dysfunction, the cytotoxicity profile of Mito6_TPP against tumor cells suggests that, instead, it might have anticancer properties.

**Figure 2:**
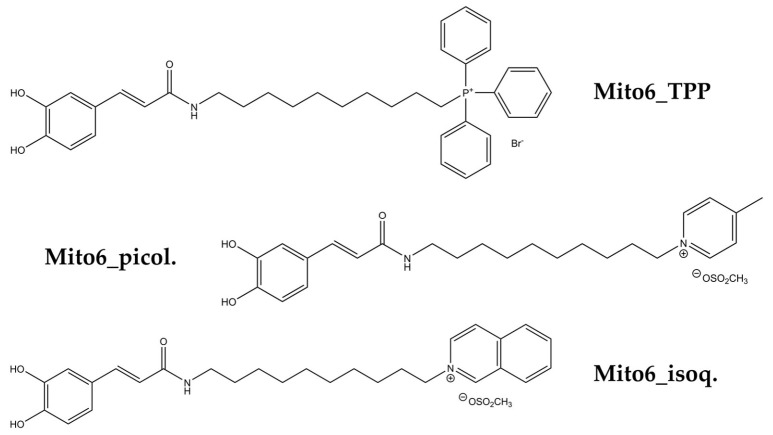
Chemical structures of mitochondria-targeted antioxidants used in this study.

This study aims to uncover the in vitro effects of potential mitocans with antioxidant properties: Mito6_TPP and two other similar compounds containing different cation carriers: 4-picolinium (Mito6_picol.) or isoquinolinium (Mito6_isoq.) (Figure 2). The compounds were tested in cell lines of SCCOHT and type 1 and type 2 endometrial cancer, as the treatment for these pathologies is mainly based on cytotoxic drugs and there is a need to find targeted drugs to treat them. The results of this study will demonstrate the potential of these compounds as anticancer agents that induce tumor cell death, as well as their selectivity for tumor cells.

## 2. Materials and Methods

### 2.1. Reagents

All reagents and cell culture material were of analytical grade or of the highest grade available and were acquired from Sigma-Aldrich Co. (St. Louis, MO, USA). Antibiotic-antimycotic (AB-AM) was acquired from Grisp (Porto, Portugal), Trypsin/EDTA 2.5%, 3,3′-Dihexyloxacarbocyanine Iodide (DiOC_6_) from Gibco/Invitrogen Co. (Carlsbad, CA, USA), Pierce Lactate Dehydrogenase (LDH) cytotoxicity assay kit from Thermo Fisher (Waltham, MA, USA), and Dibutylphthalate Polystyrene Xylene (DPX) from VWR-Prolabo (Radnor, PA, USA). Culture flasks were from Sarstedt (Nümbrecht, Germany) and all the other plastic materials used in cell culture techniques were either from Falcon (Tewksbury, MA, USA) or Nerbe plus (Winsen, Germany).

### 2.2. Chemistry

In an effort to discover new mitochondria-targeted antioxidants acting as anticancer agents, three mitochondriotropic antioxidants based on caffeic acid scaffolds (Mito6_TPP, Mito6_picol., and Mito6_isoq.) were synthetized de novo (Figure 2). The synthetic methodologies and the spectroscopic characterization data (nuclear magnetic resonance spectroscopy and mass spectrometry) of the compounds were performed. The compounds’ solvent was dimethyl sulfoxide (DMSO) and it was always used in further experiments at a maximum concentration of 1% *v*/*v*.

### 2.3. Cell Culture Conditions

Experiments were performed using the following cell lines: Ishikawa, Hec50co, COV434, and HFF-1. The Ishikawa cell line (RRID: CVCL_2529) was established from an endometrial adenocarcinoma [40,41]. The Hec50co cell line (RRID: CVCL_4Y59) consists of poorly differentiated endometrial tumor cells isolated from a patient with advanced disease [41]. Both the Ishikawa and Hec50co cell lines were kindly provided by Dr. Kim K. Leslie (The University of New Mexico Health Sciences Center, Albuquerque, NM, USA). COV434 (RRID: CVCL_2010) were initially isolated from a patient diagnosed with a granulosa cell tumor but were recently classified as cells from a small-cell carcinoma of the ovary, hypercalcemic type [42].The COV434 cells were purchased from the European Collection of Authenticated Cell Cultures (ECACC) (#07071909). HFF-1 (RRID: CVCL_3285) is an immortalized human foreskin fibroblasts cell line, obtained from the American Type Culture Collection (ATCC) [43], which was used as control, as they are non-tumorous cells. The Ishikawa and Hec50co cells were maintained in Dulbecco’s Modified Eagle Medium/F12 (DMEM/F12) supplemented with 5% Foetal Bovine Serum (FBS). COV434 cells were maintained in DMEM/F12 supplemented with 10% FBS_._ HFF-1 were maintained in Roswell Park Memorial Institute 1640 (RPMI 1640) supplemented with 10% FBS. All mediums were supplemented with 1% AB-AM and all cells were kept at 37 °C with 5% CO_2_. The treatment with the compounds was done in the respective cell medium supplemented with 2% FBS and 24 h after platting the cells in the culture medium with 5% FBS to allow their adherence.

### 2.4. Cell Viability and Cytotoxicity Assays

The Ishikawa, Hec50co, COV434, and HFF-1 cell lines were plated at a density of 5 × 10^4^ cells/well in transparent 96-well plates. After 24 h, the cells were treated with Mito6_TPP, Mito6_picol., or Mito6_isoq. (0.01–100 μM) and incubated at 37 °C with 5% CO_2_ for 24, 48, or 72 h. The cells were also exposed to the highest concentration of the compound’s vehicle (1% DMSO) and showed no differences compared to the control wells containing only cell medium and 2% FBS. The conditions of the methylthiazolyldiphenyl-tetrazolium bromide (MTT) assay were adapted from the work of Banerjee et al. [44]. Briefly, MTT was added to a final concentration of 0.5 mg/mL, and the cells were incubated for 3 h at 37 °C with 5% CO_2_. After the 3 h, purple MTT formazan crystals were dissolved by agitation for 15 min in a solution of DMSO/isopropanol (ratio 3:1) and quantified spectrophotometrically at 540 nm. The neutral red (NR) assay conditions were based on the work developed by Repetto et al. [45]. Briefly, after the incubation time, cells were incubated with NR at 40 μg/mL in an FBS-free cell medium for 3 h. The cells were then incubated with a solution of 50% ethanol (96% *v*/*v*), 49% deionized water, and 1% glacial acetic acid, with shaking for 15 min. The NR extracted from the cells was measured at 540 nm. The LDH cytotoxicity assay was not performed on the HFF-1 cells due to the lack of evidence of cell death at the concentrations tested. After the incubation time, this assay was performed using a Thermo Scientific Pierce LDH Cytotoxicity Assay Kit according to the manufacturer’s instructions [46]. For all assays, the absorbance was read using a BioTek Synergy HTX Multi-Mode Microplate Reader equipped with BioTek Gen5 Data Collection and Analysis Software (BioTek Instruments, Winooski, VT, USA).

### 2.5. Cell Morphology Evaluation

Morphological changes in all cell lines were evaluated by phase-contrast microscopy (Eclipse TS100 Inverted Microscope, Nikon, Tokyo, Japan) and Giemsa staining. Additionally, nuclear changes were evaluated by Höechst staining only in the Ishikawa, Hec50co, and COV434 cell lines, as a lack of evidence of cell death was observed in HFF-1 cells. The cells were plated in 24-well plates with coverslips using the densities of 1 × 10^5^ cells/well in Ishikawa and Hec50co and 2.5 × 10^5^ cells/well in COV434 and HFF-1. After adherence, the cells were treated with Mito6_TPP (5 μM), Mito6_picol. (20 μM), or Mito6_isoq. (10 μM) and incubated at 37 °C with 5% CO_2_ for 72 h. For Giemsa staining [47], the cells were fixated with paraformaldehyde 4% in Phosphate Buffered Saline (PBS) for 15 min, stained with Giemsa solution for 30 min, mounted with DPX, and observed through bright field microscopy. For Höechst staining [48], the cells were incubated with 0.5 μg/mL Höechst 33342 in PBS for 20 min in the dark, mounted in Fluoroshield, and observed under a fluorescence microscope equipped with an excitation filter with maximum transmission at 360/400 nm. In both stainings, the cells were observed using the microscope Eclipse CI (Nikon, Japan) and the images were processed with Nikon NIS Elements Image Software (Nikon, Japan).

### 2.6. Mitochondrial Membrane Potential (Δψm) Assessment

The Ishikawa, Hec50co, COV434, and HFF-1 cell lines were plated at a density of 5 × 10^4^ cells/well in black 96-well plates. After 24 h, the cells were treated with Mito6_TPP (5 μM), Mito6_picol. (20 μM), or Mito6_isoq. (10 μM) and incubated at 37 °C with 5% CO_2_ for 48 h. The Δψm study was adapted from the work of Sivandzade et al. [49]. The mitochondrial membrane-depolarizing agent carbonyl cyanide m-chlorophenylhydrazone (CCCP) (50 μM) was used as the positive control, being added to the cells for 20 min prior to the addition of the probe DiOC_6_ (100 nM) for 30 min at 37 °C in the dark. The fluorescence was then measured using a BioTek Synergy HTX Multi-Mode Microplate Fluorimeter equipped with BioTek Gen5 Data Collection and Analysis Software (BioTek Instruments, Winooski, VT, USA) (excitation: 488 nm; emission: 525 nm).

### 2.7. Intracellular Reactive Oxygen and Nitrogen Species (ROS/RNS) Assessment

The assay for the evaluation of the levels of ROS/RNS was based on the work of Bak, et al. [50]. All cell lines were cultured in black 96-well plates at a density of 5 × 10^4^ cells/well and allowed to adhere for 24 h. After adherence, the cells were incubated with 2′,7′-dichlorodihydrofluorescein diacetate (DCFH-DA) (25 μM) for 1 h at 37 °C in the dark. The DCFH-DA probe allows the detection of intracellular ROS, more specifically H_2_O_2_, HO^●^ and ROO^●^, and RNS, more specifically, ^●^NO and ONOO^−^ [51]. The cells were then treated with 10 μM of either Mito6_TPP, Mito6_picol., or Mito6_isoq. The stress inducer H_2_O_2_ (200 μM) was used as the positive control. The fluorescence provided by the probe was measured immediately and every 5 min for 2 h at 37 °C using a BioTek Synergy HTX Multi-Mode Microplate Fluorimeter equipped with BioTek Gen5 Data Collection and Analysis Software (BioTek Instruments, Winooski, VT, USA) (excitation: 485 nm; emission: 530 nm).

### 2.8. Statistical Analysis and Software

Data were evaluated using the one- or two-way analysis of variance (ANOVA) test followed by Bonferroni’s test for multiple comparisons. The data presented was expressed as mean ± SEM (Standard Error of the Mean) of a minimum of three and a maximum of seven independent experiments performed in triplicate. Values of *p* < 0.05 were considered statistically significant. Half of the maximal effective concentration (EC50) values were estimated by interpolation of dose-response curves and reported as a mean with a 95% confidence interval (CI 95%). All statistical analysis was performed using GraphPad Prism Software 8.0 (GraphPad Software, Inc., San Diego, CA, USA). Structure properties of the compounds were calculated using MarvinSketch 20.9 with the Calculator Plugins (ChemAxon, Budapest, Hungary). All images were created using Inkscape 1.1.1. and chemical structures were drawn using ChemDraw 19.1. (PerkinElmer, Waltham, MA, USA).

## 3. Results

### 3.1. Mito6_TPP, Mito6_picol., and Mito6_isoq. Induce Decrease in Tumor Cells’ Viability at Low Concentrations

**Figure 3:**
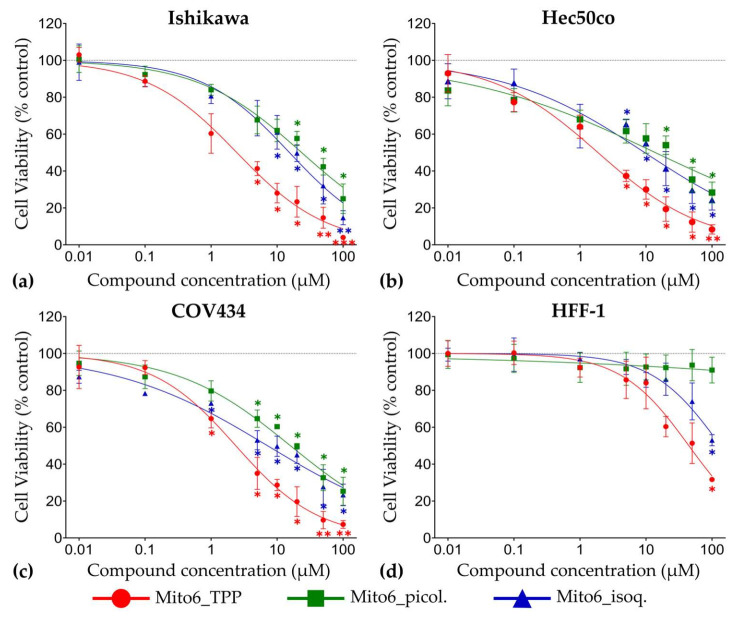
Viability assessed by the methylthiazolyldiphenyl-tetrazolium bromide (MTT) assay of Ishikawa (**a**), Hec50co (**b**), COV434 (**c**), and HFF-1 (**d**) cells treated with Mito6_TPP, Mito6_picol., or Mito6_isoq. (0.01–100 μM) at 48 h of incubation. Untreated cells containing only the respective cell medium and 2% Foetal Bovine Serum (FBS) were used as control and are represented as a dotted line at 100%. Results are compared to the control and expressed as mean ± SEM of at least three independent experiments performed in triplicate. Significant differences between treated and untreated cells are described as * (*p* < 0.05), ** (*p* < 0.01), and *** (*p* < 0.001).

**Figure 4:**
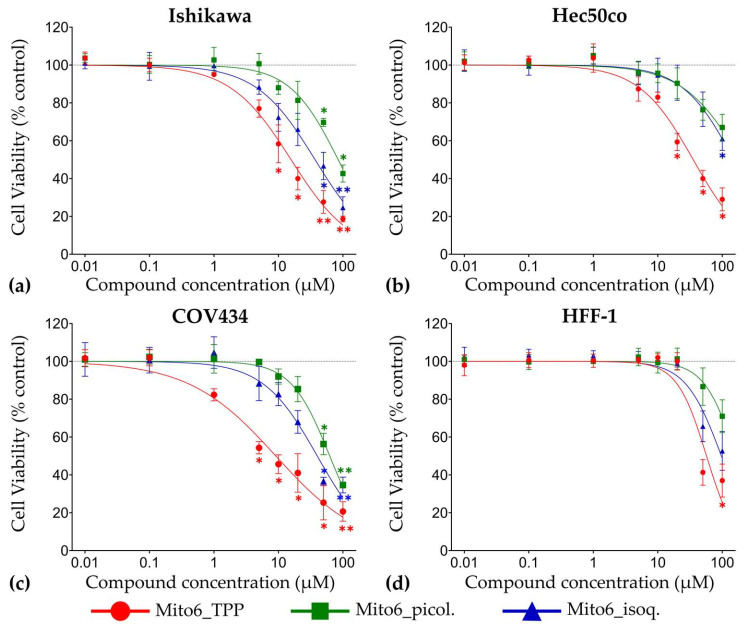
Viability assessed by the Neutral Red (NR) assay of Ishikawa (**a**), Hec50co (**b**), COV434 (**c**), and HFF-1 (**d**) cells treated with Mito6_TPP, Mito6_picol., or Mito6_isoq. (0.01–100 μM) at 48 h of incubation. Untreated cells containing only the respective cell medium and 2% FBS were used as control and are represented as a dotted line at 100%. Results are compared to the control and expressed as mean ± SEM of at least three independent experiments performed in triplicate. Significant differences between treated and untreated cells are described as * (*p* < 0.05) and ** (*p* < 0.01).

All compounds were able to decrease the metabolic activity (Figure 3) and lysosomal activity (Figure 4) after 48 h in Ishikawa, Hec50co, COV434, and HFF-1 cells in a concentration-dependent manner. Moreover, their effects were also dependent on the time of incubation (Figures S1 and S2). In all cell lines, Mito6_TPP was the most effective of the three compounds tested, as it induced a significant decrease in cell viability at concentrations as low as 1 μM. The compound Mito6_isoq. was the second most potent compound, and the least effective compound was Mito6_picol., which always required higher concentrations than the other two compounds to induce a decrease in cell viability. Overall, COV434 were the most affected cells, as they were affected by lower concentrations of the compounds than the other tumor cell lines tested. There was no clear difference in the sensitivity to the compounds between Ishikawa and Hec50co, as depending on the compound and its incubation time, the most affected of these cell lines varied. Moreover, the HFF-1 cells only experienced effects at the highest concentrations tested and were always more affected by Mito6_TPP than by the other two compounds. As the tumor cell lines always suffered a higher effect than the non-tumor cells, it is noticeable that the compounds might have selectivity for this type of cells.

The cytotoxicity was evaluated using the LDH release assay in Ishikawa, Hec50co, and COV434; however, it was not conducted on HFF-1 cells, as the previous assays showed no signs of cell viability decrease. The results also confirmed the concentration-dependent effect, supporting the previously obtained results from the MTT and NR assays (Figure S3). In addition, these results were helpful in determining the concentrations to be used in the following assays, as it allowed choosing the intermediate concentrations where there was a cell viability decrease but not cell lysis. Accordingly, Mito6_TPP, Mito6_picol., and Mito6_isoq were used in concentrations of 5 μM, 20 μM, and 10 μM, respectively. It is also relevant to point out that Mito6_isoq. was able to induce the same effects on cell viability as those of Mito6_TPP if a higher incubation time was used, but without inducing cell lysis at the same concentration.

The IC50 values were not calculated for these compounds, as there is still no clear evidence of their mechanism of action. Therefore, only the EC50 values were calculated, as it is only known that they have the capacity of inducing cell viability decrease. These results are present in Table 1. Although the values obtained through the MTT and the NR assays are different, they show the same tendency of decreasing cellular viability in a time- and concentration-dependent manner (Table 1, Tables S1 and S2). As the values obtained for the HFF-1 cells were always higher than the ones obtained for the tumor cell lines, this suggests, once again, the selectivity of the compounds to this type of cells.

### 3.2. Mito6_TPP and Mito6_isoq. Induce Alterations in Cell Morphology

**Figure 5:**
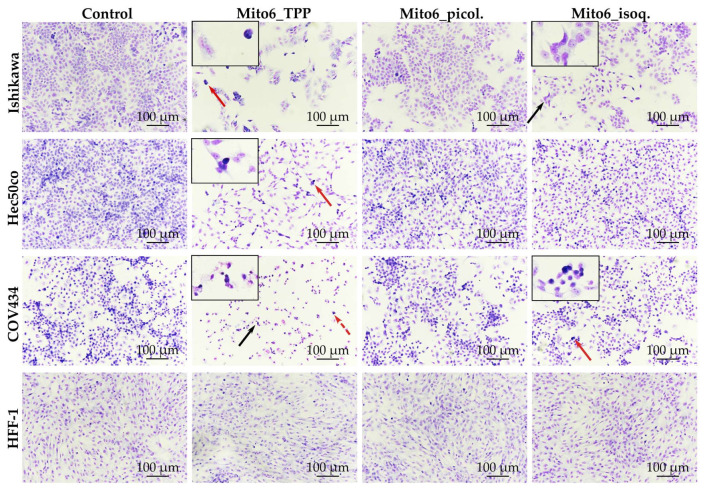
Cell morphology assessed by Giemsa staining of Ishikawa, Hec50co, COV434, and HFF-1 cells after incubation with 5 μM of Mito6_TPP, 20 μM of Mito6_picol., or 10 μM of Mito6_isoq. Untreated cells containing only the respective cell medium and 2% FBS were used as control. The results presented are representative of at least three independent experiments. Black arrows indicate examples of abnormal cell morphology and red arrows indicate examples of chromatin condensation. The black boxes contain higher magnification images containing the cell morphology or chromatin condensation indicated by the full arrows. The traced arrows are not in the enlarged box.

The study of cell morphology allowed us to notice the decrease in cell numbers (Figure 5). Moreover, all tumor cell lines showed altered cell shapes and indications of chromatin condensation when incubated with Mito6_TPP, which was already demonstrated to be the most potent compound tested. When incubated with Mito6_isoq., the Ishikawa and COV434 cells presented cell shape alterations, and COV434 even showed indications of chromatin condensation. Mito6_picol. did not induce any morphological changes in any cell line. Regarding HFF-1 cells, none of the compounds induced changes in their morphology. In fact, these results support the hypothesis that the compounds tested effectively display selectivity to tumor cells.

### 3.3. Mito6_TPP and Mito6_isoq. Induce Chromatin Condensation and Nuclear Fragmentation

**Figure 6:**
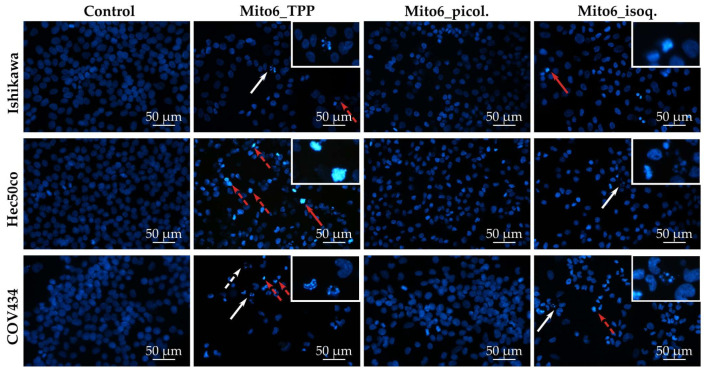
Cell morphology assessed by Höechst staining of Ishikawa, Hec50co, COV434, and HFF-1 cells after incubation with 5 μM of Mito6_TPP, 20 μM of Mito6_picol., or 10 μM of Mito6_isoq. Untreated cells containing only the respective cell medium and 2% FBS were used as control. The results presented are representative of at least three independent experiments. White arrows indicate examples of nucleus fragmentation and red arrows indicate examples of chromatin condensation. The white boxes contain higher magnification images containing the nucleus fragmentation or chromatin condensation indicated by the full arrows. The traced arrows are not in the enlarged box.

To further explore the chromatin condensation suggested by the Giemsa staining, the Höechst staining was performed and allowed to confirm its presence (Figure 6). In more detail, it was possible to observe changes in the nucleus shape and the chromatin condensation in all tumor cell lines upon incubation with Mito6_TPP or Mito6_isoq. In addition, all tumor cell lines presented further signs of cell death, as there was nuclear fragmentation visible when incubated with Mito6_TPP or Mito6_isoq. Once again, Mito6_picol. did not induce any morphological changes in any cell line. Overall, as expected due to the previous data, the COV434 cells demonstrated more signs of cell death, compared to the other two tumor cell lines. The HFF-1 cells were not stained with Höechst, as the aim was to observe possible cell death and there were no previous indications of a cell viability decrease in these cells at the concentrations tested.

### 3.4. Mito6_TPP and Mito6_isoq. Induce Reduction of Mitochondrial Membrane Potential (Δψm)

**Figure 7:**
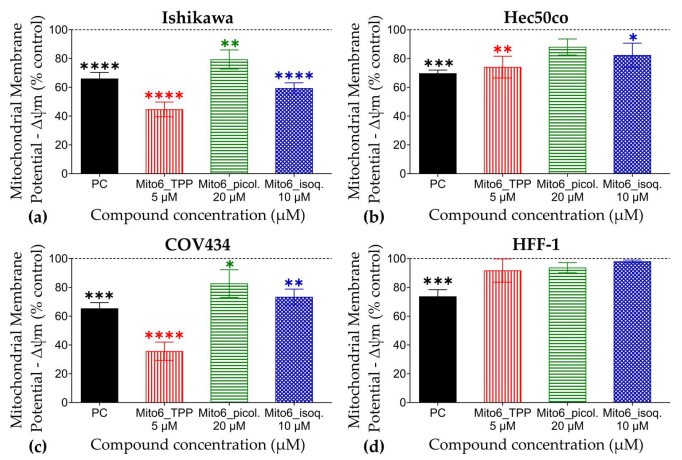
Mitochondrial membrane potential (Δψm) of Ishikawa (**a**), Hec50co (**b**), COV434 (**c**), and HFF-1 (**d**) cells after 48 h of incubation with 5 μM of Mito6_TPP, 20 μM of Mito6_picol., or 10 μM of Mito6_isoq. Untreated cells containing only the respective cell medium and 2% FBS were used as control and are represented as a dotted line at 100%. Carbonyl cyanide m-chlorophenylhydrazone (CCCP) (50 μM) was used as the positive control (PC). Results are compared to the control and expressed as mean ± SEM of at least three independent experiments performed in triplicate. Significant differences between treated and untreated cells are described as * (*p* < 0.05), ** (*p* < 0.01) and *** (*p* < 0.001), and **** (*p* < 0.0001).

The Δψm measurement showed that both Mito6_TPP and Mito6_isoq. were able to induce a significant decrease in the Δψm of all tumor cell lines, although Mito6_TPP produced more drastic effects (Figure 7). Mito6_picol. induced a decrease only in the Ishikawa and COV434 cells. Regarding HFF-1, none of the compounds induced effects, which is in accord with the previous findings. These results support the previous results of the Giemsa and Höechst staining, which indicate the possibility of the existence of apoptosis at these concentrations and, once more, the selectivity of the compounds to tumor cells.

### 3.5. Mito6_TPP, Mito6_picol. and Mito6_isoq. Induce a Reduction of Intracellular ROS

**Figure 8:**
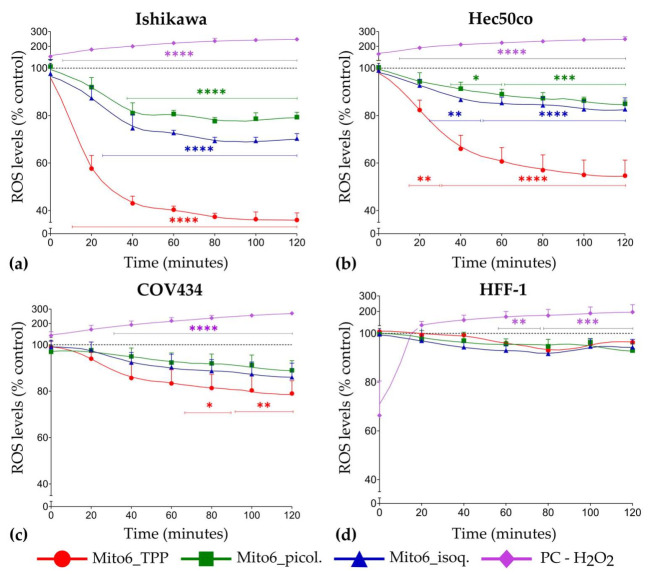
Intracellular reactive oxygen species (ROS) levels in Ishikawa (**a**), Hec50co (**b**), COV434 (**c**), and HFF-1 (**d**) cells in the 2 h immediately after the addition of 10 μM of Mito6_TPP, Mito6_picol., or Mito6_isoq. Untreated cells containing only the respective cell medium and 2% FBS were used as control and are represented as a dotted line at 100%. H_2_O_2_ (200 μM) was used as the positive control (PC). Results are compared to the control and expressed as mean ± SEM of at least three independent experiments performed in triplicate. Significant differences between treated and untreated cells are described as * (*p* < 0.05), ** (*p* < 0.01) and *** (*p* < 0.001), and **** (*p* < 0.0001).

A decrease in intracellular ROS generation (Figure 8) was detected in the tumor cells when incubated with the compounds in comparison to untreated cells. Mito6_TPP was the most effective compound, as it induced a faster and more accentuated decrease in ROS levels in all tumor cell lines, followed by Mito6_isoq. and Mito6_picol., which had no effects in COV434 cells. This result is in accord with the previous results. In addition, it supports the hypothesis that Mito6_TPP is the most potent compound studied. Surprisingly, COV434 was the less affected cell line regarding the intracellular ROS and, as expected, HFF-1 cells did not experience any effect when incubated with any of the mitochondria-targeted antioxidants. Moreover, this drastic decrease in ROS levels in the presence of the studied compounds may be related to the decrease in cell viability, as the tumor cells stop having the necessary amount of ROS they need to proliferate [24].

### 3.6. Mito6_picol. and Mito6_isoq. Comply with Lipinski’s Rules for Oral Bioavailability

As the hydrophobic character of a compound is an important factor for its bioavailability, a theoretical study about the chemical properties of all tested compounds was made and compared to Lipinski’s and Veber’s rules for good oral bioavailability of drugs. Lipinski’s rule of five states that orally absorbed drugs usually have a molecular weight lower than 500 g/mol, less than 5 hydrogen-bond donors, less than 10 hydrogen-bond acceptors, and a logP minor than +5 [52]. Veber’s rules state that the compounds should have a polar surface area lower than 140 Å and no more than 10 rotatable bonds [52]. The values present in Table 2 indicate that Mito6_TPP does not comply with half of Lipinski’s and Veber’s rules, while Mito6_picol. and Mito6_isoq. do not comply with only one of Veber’s rules and comply with all of Lipinski’s requirements. Although these rules are only guidelines and there are a lot of commercially available drugs that do not comply with these rules, these results give an indication that Mito6_picol. and Mito6_isoq. might be more likely to have a good oral bioavailability than Mito6_TPP. However, it is also important to observe the high value of logP of Mito6_TPP that indicates the high lipophilicity of the compound, which may contribute to its ability to enter and accumulate within the mitochondria.

## 4. Discussion

Considering the assays performed in this study, the three compounds tested were able to induce a reduction of cell viability in tumor cells. Nevertheless, Mito6_TPP was the most effective one, as it dramatically reduced cell viability even at low concentrations and incubation times. Regarding the other two compounds, Mito6_isoq. produced lower effects than Mito6_TPP but higher than Mito6_picol. Additionally, the effects of the compounds were different depending on the cell line. COV434 was always the most affected tumor cell line. Regarding HFF-1 non-tumor cells, they always needed a higher concentration of any of the compounds, compared with the tumor cells, to exert any effects. This proves that the three compounds studied had a greater tendency to target tumor cells instead of affecting all cells indiscriminately. There were also indications that the mechanism of death induced by the compounds, at the concentrations tested, is related to selective apoptotic events, due to the presence of chromatin condensation and nucleus fragmentation and a decrease in the mitochondrial membrane potential. All these differences between the effects of the compounds in the different cell lines derive from the diverse characteristics and metabolic pathways of the cells that will affect their response to the compounds. 

In this particular case, the fact that the mitochondria membrane potential in tumor cells is highly different than in non-tumorous cells will enhance the uptake of the compounds that contain a lipophilic cation carrier, which can help explain their preference to the tumor cell lines. Moreover, the higher quantity of ROS that tumor cells contain, compared to non-tumorous cells, will stimulate the increase of activity of the antioxidants. It is not yet totally clear whether Mito6_TPP or Mito6_isoq. has more potential. This is due to the fact that Mito6_isoq. always needs more incubation time to achieve the same effects that Mito6_TPP can produce, but it has the advantage that concentrations inferior to 100 μM do not induce any changes in non-tumor cells. Overall, Mito6_TPP is capable of affecting the tumor cells more rapidly, but Mito6_isoq. is able to do it more selectively. Additionally, Mito6_isoq. failed only one of Lipinski’s and Veber’s rules, implying that, theoretically, it has a greater potential to be orally bioactive than Mito6_TPP, which only complied with half of them; however, these rules are only guidelines and are not followed by many available drugs.

Although the exact mechanism of action of these compounds is not yet determined, the results here presented demonstrate their potent mitochondriotropic redox properties. The compounds might be participating in a simple redox reaction, as they may directly interact with ROS inside the mitochondria, or they may be inhibiting one of the enzymes responsible for the ROS production. Regarding the mechanism of the compounds’ delivery to the cells, it is strongly dependent on the type of cation carrier, with the TPP being considered the most effective cation among the three that were tested. The potency of the TPP cation in comparison with the other two cations can have multiple explanations, one of them being the fact that the TPP cation confers to the compound a more lipophilic character, as shown by the logP calculation, which can contribute to its capacity to accumulate in the mitochondria. Moreover, it can also possibly be associated with the protonophore effect that was previously related in some compounds conjugated with TPP [53,54,55]. This effect can induce a reduction of the Δψm and also a reduction of the production of ROS by the mitochondria; however, this theory still needs further studies. Additionally, the dramatic decrease observed in intracellular ROS levels might be a cause of the decrease in tumor cell viability, since the cells are losing the necessary quantity of ROS they need to maintain their normal signaling processes and will stop proliferating [24]. The treatment of tumor cells with this type of mitochondria-targeted antioxidants will induce a decrease in ROS levels and cells will not have the necessary quantity of ROS they need to maintain their growth. However, as tumor cells are highly dependent on the balance between ROS and antioxidants, the increase of ROS in a high enough way that it exceeds the antioxidant capacity of the cell is also a potent anticancer strategy that is already being used as a treatment [56]. The use of lipophilic cations, such as the ones used in this study, might also improve that strategy, as it can help direct the compounds to the mitochondria and more efficiently stimulate ROS production. Some studies using the TPP cation conjugated with various families of molecules have also been conducted in that way and have demonstrated anticancer potential by inducing an increase in ROS [57,58,59].

Overall, all the compounds tested demonstrated the potential to act as mitotargeted anticancer agents against a rare tumor such as SCCOHT and against type 1 and type 2 endometrial tumors, especially Mito6_TPP and Mito6_isoq. They can bring several advantages compared to the current treatment applied to these pathologies. The main advantage is the fact that these compounds are able to select between tumor and non-tumor cells and target mainly the tumor cells, which may drastically reduce the side effects known to be attached to cytotoxic drugs. As promising as these compounds seem, they still need to be deeply studied to determine their exact mechanism of action, their bioavailability, their metabolization profile, and other effects they may cause in a complex organism. Even if further studies disprove the potential of these compounds, they may still be a basis for the development of new mitochondria-targeted antioxidants that can act as mitocans.

## 5. Conclusions

In general, this study demonstrates the capacity of the compounds tested to selectively induce a decrease in the cell viability of tumor cells. In particular, both Mito6_TPP and Mito6_isoq. showed great potential for combating SCCOHT and type 1 and type 2 endometrial tumors, while barely affecting non-tumor cells. Moreover, the compounds’ target is the mitochondria that are constitutively present in all tumors, which may allow for the consideration of these compounds as solutions for treatments of other tumors, even though they can have their biggest effect in tumors that are highly dependent on the oxidative metabolism. Although the mechanisms underlying the effects of these compounds need to be deeply studied, overall this study supports the development of new mitochondria-targeted antioxidants that can act as mitocans and substitute, and/or complement, current cancer therapies.

## 6. Patents

Mitochondria-targeted antioxidants, processes, and applications are under patent (PCT/IB2017/056412; US 2019/0248816 A1).

## Figures and Tables

**Table 1:** The EC50 values of Mito6_TPP, Mito6_picol., and Mito6_isoq. in Ishikawa, Hec50co, COV434, and HFF-1 cells at 48 h of incubation. The values were calculated by interpolation in GraphPad Prism using the decrease of viability obtained through the MTT or the NR assay.

48 h	EC50 (μM)–Mean (CI 95%) ^1^					
	MTT Assay			NR Assay		
	Mito6_TPP	Mito6_picol.	Mito6_isoq.	Mito6_TPP	Mito6_picol.	Mito6_isoq.
Ishikawa	2.453	23.56	15.39	15.69	84.44	36.94
	(1.859–3.188)	(18.36–30.69)	(11.49–20.39)	(13.47–18.29)	(70.97–105.2)	(31.15–44.21)
Hec50co	1.872	13.82	9.554	35.41	197.3	150.4
	(1.355–2.530)	(8.240–24.37)	(6.105–14.78)	(30.32–41.67)	(139.5–350.2)	(111.7–251.7)
COV434	2.367	15.89	7.156	9.107	62.46	39.97
	(1.802–3.054)	(12.31–20.53)	(5.021–10.07)	(7.122–11.58)	(56.43–69.54)	(32.63–49.84)
HFF-1	44.54	>100	>100	56.98	>100	96.96
	(34.53–59.93)	---	---	(47.18–70.11)	---	(81.95–121.3)

^1^ CI: Confidence Interval at 95%.

**Table 2:** Theoretical chemical properties of the compounds and their compliance to Lipinski’s and Veber’s rules for oral bioavailability. The values were calculated using MarvinSketch 20.9 with the Calculator Plugins.

	Lipinski’s Rules					Veber’s Rules		
	MW	logP	HBA	HBD	Lipinski’s Violations	PSA	NRB	Veber’s Violations
	≤500	≤+5	≤10	≤5		≤140	≤10	
Mito6_TPP	580.728	8.65	3	3	2	69.56	16	1
Mito6_picol.	411.565	1.34	3	3	0	73.44	13	1
Mito6_isoq.	447.598	1.82	3	3	0	73.44	13	1

MW: Molecular Weight (g/mol); logP: Partition Coefficient; HBA: Hydrogen-Bond Acceptor Atoms; HBD: Hydrogen-Bond Donor Atoms; PSA: Polar Surface Area (Å); NRB: Number of Rotatable Bonds.

## Data Availability

Not applicable.

## Supplementary Materials

The following materials are available online at https://www.mdpi.com/article/10.3390/biomedicines10040800/s1, Figure S1: Viability of cells treated with the compounds for 24 and 72 h, assessed by the MTT assay, Figure S2: Viability of cells treated with the compounds for 24 and 72 h, assessed by the NR assay, Figure S3: Cytotoxicity of all compounds in Ishikawa, Hec50co and COV434, assessed by LDH release assay, Table S1: EC50 of all compounds in the cells at 24 h of incubation, assessed by the MTT and NR assays, and Table S2: EC50 of all compounds in the cells at 72 h of incubation, assessed by the MTT and NR assays.

## Abbreviations

AB-AM = Antibiotic-antimycotic; CCCP = Carbonyl Cyanide m-Chlorophenylhydrazone; DCFH-DA = 2′,7′-Dichlorodihydrofluorescein Diacetate; DiOC_6_ = 3,3′-Dihexyloxacarbocyanine Iodide; DMEM/F12 = Dulbecco’s Modified Eagle Medium/F12; DMSO = Dimethyl Sulfoxide; DPX = Dibutylphthalate Polystyrene Xylene; EDTA = Ethylenediamine Tetraacetic Acid; FBS = Foetal Bovine Serum; HBA = Hydrogen-Bond Acceptor Atoms; HBD = Hydrogen-Bond Donor Atoms; LDH = Lactate Dehydrogenase; logP = Partition Coefficient; MTT = Methylthiazolyldiphenyl-tetrazolium Bromide; MW = Molecular Weight (g/mol); NR = Neutral Red; NRB = Number of Rotatable Bonds; PC = Positive Control; PBS = Phosphate Buffered Saline; PSA = Polar Surface Area (Å); RNS = Reactive Nitrogen Species; ROS = Reactive Oxygen Species; RPMI = Roswell Park Memorial Institute 1640; SEM = Standard Error of the Mean; TPP = Triphenylphosphonium; Δψm = Mitochondrial membrane potential.
