# Supplementary material for: In Vitro Effects of Mitochondria-Targeted Antioxidants in a Small-Cell Carcinoma of the Ovary of Hypercalcemic Type and in Type 1 and Type 2 Endometrial Cancer
Source: Biomedicines. 2022 Mar 29;10(4):800. doi: 10.3390/biomedicines10040800 (PMC9030827; doi:10.3390/biomedicines10040800)
Supplement: Supplementary file 1 [file biomedicines-10-00800-s001.zip › biomedicines-1622807-supplementary.pdf]

biomedicines

Supplementary Materials for

In Vitro Effects of Mitochondria-Targeted Antioxidants in a Small-Cell Carcinoma of the Ovary of Hypercalcemic Type and in Type 1 and Type 2 Endometrial Cancer

Mariana Castelôa [1], Beatriz Moreira-Pinto [2,3], Sofia Benfeito [1], Fernanda Borges [1], Bruno M. Fonseca [2,3,*], and Irene Rebelo [2,3,*]

[1] CIQUP/Department of Chemistry and Biochemistry, Faculty of Sciences, University of Porto, Rua do Campo Alegre 1021/1055, 4169-007 Porto, Portugal; mariana15cas@gmail.com (M.C.) ester.benfeito@fc.up.pt (S.B.); fborges@fc.up.pt (F.B.)
[2] UCIBIO, Applied Molecular Biosciences Unit, Laboratory of Biochemistry, Faculty of Pharmacy, University of Porto, Rua de Jorge Viterbo Ferreira 228, 4050-313 Porto, Portugal; abeatrizmoreira.pinto@gmail.com (B. M.-P.)
[3] Associate Laboratory i4HB, Institute for Health and Bioeconomy, Laboratory of Biochemistry, Faculty of Pharmacy, University of Porto, Rua de Jorge Viterbo Ferreira 228, 4050-313 Porto, Portugal
* Correspondence: brunofonseca@ff.up.pt (B.M.F.); irebelo@ff.up.pt (I.R.)

Index

Figure S1. Viability of cells treated with the compounds for 24 and 72 h, assessed by the MTT assay……………..………..2
Figure S2. Viability of cells treated with the compounds for 24 and 72 h, assessed by the NR assay……………………..…….3
Figure S3. Cytotoxicity of all compounds in Ishikawa, Hec50co and COV434, assessed by LDH release assay…..………4
Table S1. EC50 values of all compounds in the cells at 24 h of incubation, assessed by the MTT and NR assays...………5
Table S2. EC50 values of all compounds in the cells at 72 h of incubation, assessed by the MTT and NR assays...………6

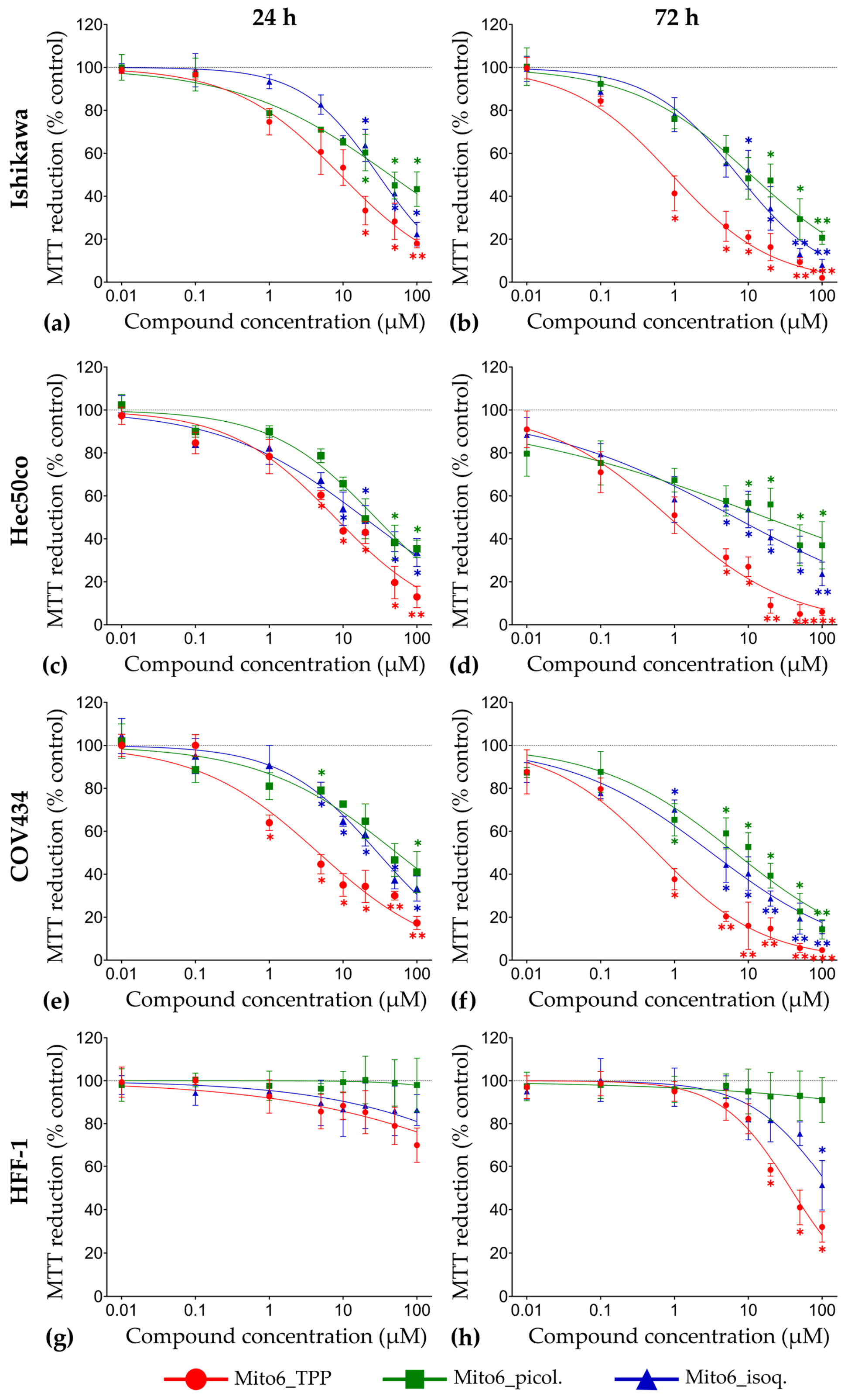

Figure S1. Viability assessed by the MTT assay of Ishikawa **(a, b)**, Hec50co **(c, d)**, COV434 **(e, f)** and HFF-1 **(g, h)** cells treated with Mito6_TPP, Mito6_picol. or Mito6_isoq. (0,01-100 µM) for 24 and 72 hours. Untreated cells were used as control and are represented as a dotted line at 100%. Results are compared to the control and expressed as mean ± SEM of at least three independent experiments performed in triplicate. Significant differences between treated and untreated cells are described as * (p<0,05), ** (p<0,01) and *** (p<0,001).

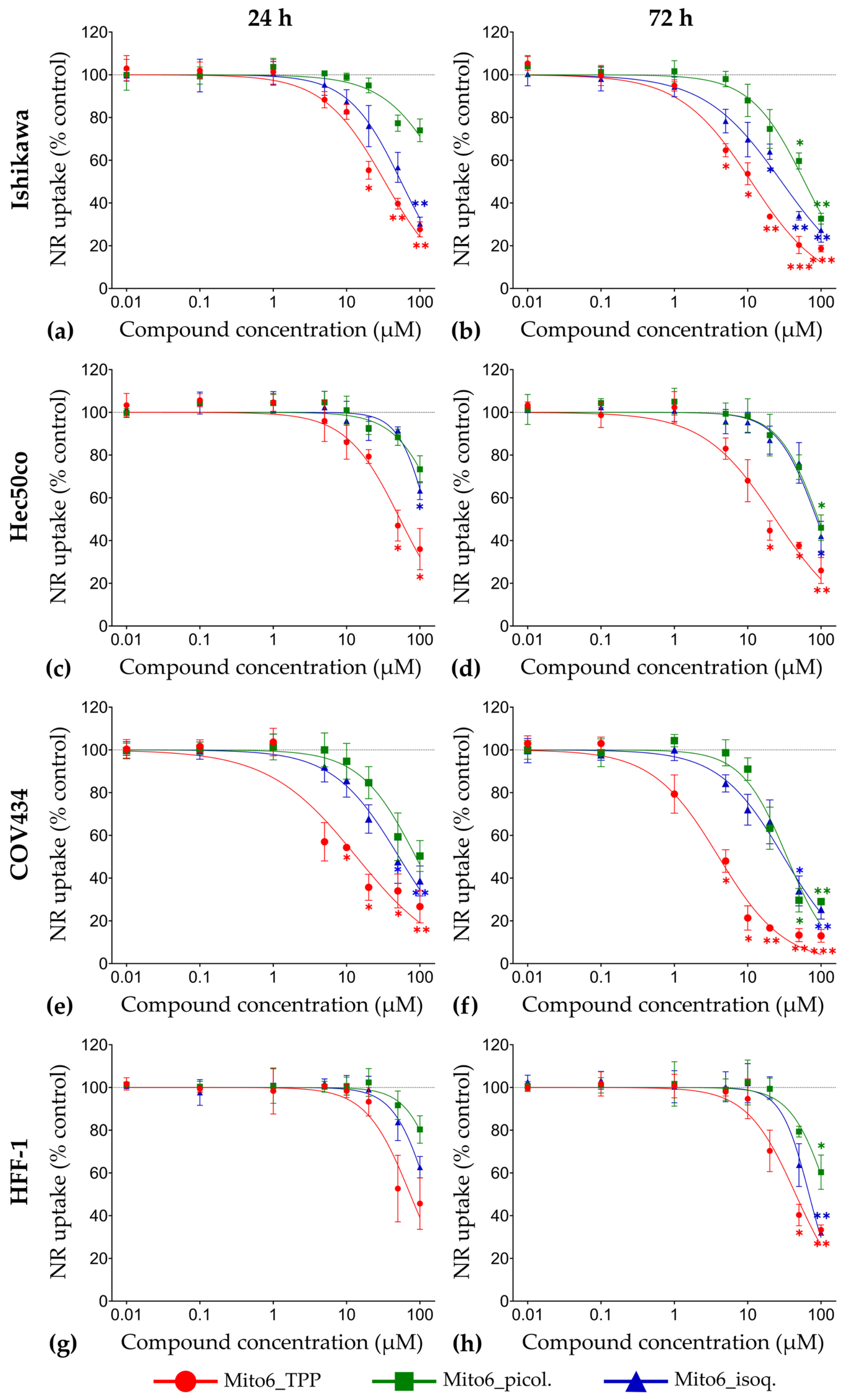

Figure S2. Viability assessed by the NR assay of Ishikawa (a, b), Hec50co (c, d), COV434 (e, f) and HFF-1 (g, h) cells treated with Mito6_TPP, Mito6_picol. or Mito6_isoq. (0,01-100 µM) for 24 and 72 hours. Untreated cells were used as control and are represented as a dotted line at 100%. Results are compared to the control and expressed as mean ± SEM of at least three independent experiments performed in triplicate. Significant differences between treated and untreated cells are described as * (p<0,05), ** (p<0,01) and *** (p<0,001).

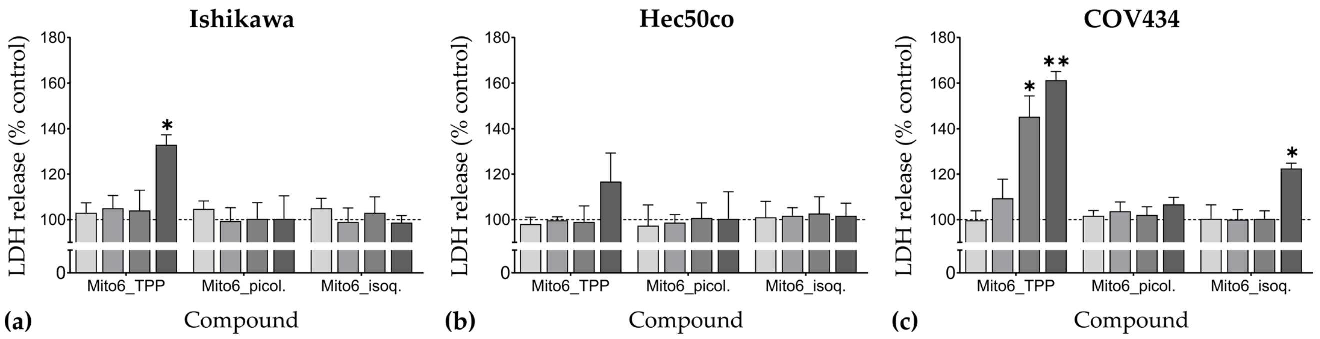

Figure S3. Cytotoxicity of Mito6_TPP, Mito6_picol. or Mito6_isoq. (1-20 μM) assessed by LDH release assay in Ishikawa **(a),** Hec50co **(b)** and COV434 **(c)** cells after 48h of incubation. Untreated cells were used as control and are represented as a dotted line at 100%. Results are compared to the control and expressed as mean ± SEM of at least three independent experiments performed in triplicate. Significant differences between treated and untreated cells are described as * (p<0,05) and ** (p<0,01).

Table S1. The EC50 values of Mito6_TPP, Mito6_picol. and Mito6_isoq. in Ishikawa, Hec50co, COV434, and HFF-1 cells at 24 hours of incubation. The values were calculated by interpolation in GraphPad Prism using the decrease of viability obtained through the MTT or the NR assay.

**24h**	**EC50 (μM) – Mean (CI 95%)[1]**					
	**MTT assay**			**NR assay**		
	**Mito6_TPP**	**Mito6_picol.**	**Mito6_isoq.**	**Mito6_TPP**	**Mito6_picol.**	**Mito6_isoq.**
Ishikawa	9.142<br>(7.047 - 11.73)	42.92<br>(31.09 - 63.27)	29.85<br>(25.36 - 3535)	33.16<br>(28.58 - 38.72)	>100<br>---	55.29<br>(48.02 - 64.32)
Hec50co	7.746<br>(5.836 - 10.07)	27.33<br>(21.57 - 35.43)	19.02<br>(14.08 - 26.23)	53.65<br>(44.62 - 65.82)	>100<br>---	>100<br>---
COV434	4.644<br>(3.317 - 6.418)	53.21<br>(36.54 - 88.56)	29.5<br>(23.61 - 37.58)	13.62<br>(9.671 - 19.38)	87.76<br>(70.73 - 117.0)	51.62<br>(42.41 - 64.49)
HFF-1	>100<br>---	>100<br>---	>100<br>---	73.62<br>(59.20 - 96.85)	>100<br>---	>100<br>---

[1]CI: Confidence Interval at 95%.

Table S2. The EC50 values of Mito6_TPP, Mito6_picol. and Mito6_isoq. in Ishikawa, Hec50co, COV434, and HFF-1 cells at 72 hours of incubation. The values were calculated by interpolation in GraphPad Prism using the decrease of viability obtained through the MTT or the NR assay.

72h	EC50 (μM) – Mean (CI 95%)[1]					
	MTT assay			NR assay		
	Mito6_TPP	Mito6_picol.	Mito6_isoq.	Mito6_TPP	Mito6_picol.	Mito6_isoq.
Ishikawa	0.9248 (0.6905 - 1.225)	10.80 (8.142 - 14.23)	7.160 (5.313 - 9.380)	11.19 (9.659 - 12.94)	59.40 (51.37 - 69.61)	28.88 (24.37 - 34.48)
Hec50co	0.8468 (0.5669 - 1.235)	17.24 (8.418 - 42.24)	6.590 (4.170 - 10.35)	23.87 (19.35 - 29.75)	92.01 (79.42 - 111.8)	87.58 (76.49 - 104.7)
COV434	0.5768 (0.4109 - 0.7963)	6.602 (4.310 - 9.753)	3.159 (2.213 - 4.391)	4.006 (3.189 - 4.957)	34.20 (28.56 - 41.32)	30.75 (25.95 - 36.68)
HFF-1	37.04 (30.84 - 45.14)	>100 ---	>100 ---	45.10 (38.10 - 53.97)	>100 ---	68.64 (64.43 - 77.04)

[1]CI: Confidence Interval at 95%.
